# Novel Preoperative Nomogram for Prediction of Futile Resection in Patients Undergoing Exploration for Potentially Resectable Intrahepatic Cholangiocarcinoma

**DOI:** 10.1038/srep42954

**Published:** 2017-02-17

**Authors:** Kwangwoo Nam, Dae Wook Hwang, Ju Hyun Shim, Tae Jun Song, Sang Soo Lee, Dong-Wan Seo, Sung Koo Lee, Myung-Hwan Kim, Ki-Hun Kim, Shin Hwang, Kwang-Min Park, Young-Joo Lee, Minkyu Han, Do Hyun Park

**Affiliations:** 1 Division of Gastroenterology, Department of Internal Medicine, Dankook University Hospital, Dankook University College of Medicine, Cheonan 31116, Korea; 2 Division of Hepatobiliary and Pancreatic Surgery, Department of Surgery, Asan Medical Center, University of Ulsan College of Medicine, Seoul 05505, Korea; 3 Division of Gastroenterology, Department of Internal Medicine, Asan Medical Center, University of Ulsan College of Medicine, Seoul 05505, Korea; 4 Division of Hepatobiliary Surgery and Liver Transplantation, Department of Surgery, Asan Medical Center, University of Ulsan College of Medicine, Seoul 05505, Korea; 5 Department of Clinical Epidemiology and Biostatistics, Asan Medical Center, University of Ulsan College of Medicine, Seoul 05505, Korea

## Abstract

Surgical resection is the treatment of choice for intrahepatic cholangiocarcinoma (IHCC). However, discrepancies between preoperative workup and intraoperative findings can occur, resulting in unexpected and unfavorable surgical outcomes. The aim of this study was to develop a feasible preoperative nomogram to predict futile resection of IHCC. A total of 718 patients who underwent curative-intent surgery for IHCC between January 2005 and December 2014 were included. The patients were divided into a training cohort (2005–2010, *n* = 377) and validation cohort (2011–2014, *n* = 341). The predictive accuracy and discriminative ability of the nomogram were determined by the concordance index and calibration curves. In multivariate analysis of the training cohort, tumor number, lymph node enlargement, presence of intrahepatic duct stones, and elevated neutrophil-to-lymphocyte ratio (NLR) (≥2.7) were independently correlated with the risk of futile resection. The predictive nomogram was established based on these factors. The concordance index of the nomogram for the training and the validation cohorts was 0.847 and 0.740, respectively. In this nomogram, the negative predictive value (128 points, probability of futile resection of 36%) in the validation cohort was 93.3%. In conclusion, our novel preoperatively applicable nomogram is a feasible method to predict futile resection of IHCC in curative-intent surgery.

Intrahepatic cholangiocarcinoma (IHCC), a bile duct neoplasm located mainly in the hepatic parenchyma, is the second most common primary liver cancer after hepatocellular carcinoma^1^. The incidence rate of IHCC in the US is 0.58/100,000^2^, and the age-adjusted mortality rate for IHCC is 0.65/100,000^3^. Although cases of IHCC remain rare in the US, incidences of these bile duct neoplasms have gradually increased in recent decades^1^. Complete surgical resection with a negative resection margin improves long-term outcomes and it is the only curative treatment option^4^. The 5-year survival rate after complete resection is 39%, compared with 5–10% in unresectable disease^1^.

Despite extensive preoperative imaging workup for potentially resectable IHCC, discrepancies between preoperative and intraoperative findings frequently occur with a significant number of cases of unresectable IHCC or gross remnant disease after exploration owing to radiologically occult metastatic disease^5^. In a recent study, the following issues were reported as the main causes of R2 resection: the unexpected presence of locally advanced tumors, multiple intrahepatic metastases in both lobes, or peritoneal seeding not clearly seen on imaging studies^5^. These can result in unnecessary surgery-related morbidity and delays in palliative treatment (e.g., chemotherapy or radiotherapy). Thus, the overall outcomes in such cases will be more serious than expected. Additional costs are also incurred, placing a further burden on patients and their families. Therefore, a thorough selection process is essential to ensure that only patients with potentially curable diseases proceed to resection.

Little is known about the ability of preoperative imaging and laboratory findings to predict futile resection of potentially resectable IHCC. Thus, a new predictive model is required to determine additional discriminators of futile resection among high-risk patients. The aim of this study was to identify preoperative predictors of futile resection at surgical exploration in patients with well-preserved liver function and potentially resectable IHCC, and then to build a patient-based practical nomogram to predict futile resection using the derived variables.

## Results

### Patients’ demographics and clinical outcomes

This study consisted of 718 patients, with 377 patients in the training cohort (Table 1). The median age was 60 years, and 260 (69.0%) patients were male. The median tumor size was 5.0 cm, and 45 (11.9%) patients had multiple tumors. The imaging studies performed at diagnosis indicated that lymph node enlargement was positive in 134 (35.5%) patients. The median neutrophil-to-lymphocyte ratio (NLR) was 2.2, and R2 resection occurred in 61 (16.2%) patients. The reasons for R2 resection were as follows: peritoneal seeding (*n* = 19), abdominal wall invasion (*n* = 12), hilar or major vessel invasion (*n* = 11), multiple intrahepatic metastases in both lobes (*n* = 10), distant lymph node metastasis (*n* = 7; aortocaval (n = 3), cardiophrenic (n = 2), paraaortic (n = 2)), and a small remnant liver volume (*n* = 2). Among them, 43 patients (70.5%) underwent laparotomy only, while resection of the lesion was precluded. None of these findings were apparent in the preoperative imaging studies.

In the univariate analysis, the presence of intrahepatic duct (IHD) stones, tumor location, tumor number, vascular invasion, lymph node enlargement, serum alkaline phosphatase, elevated NLR (≥2.7), and serum CA-19-9 level were significant predictors of futile resection. The multivariate analysis identified the presence of IHD stones (*P = *0.016), tumor number (*P* < 0.001), lymph node enlargement (*P* < 0.001), and an elevated NLR (*P* = 0.042) as independent predictors of futile resection (Table 2).

### Nomogram of the training cohort

**Figure 1:**
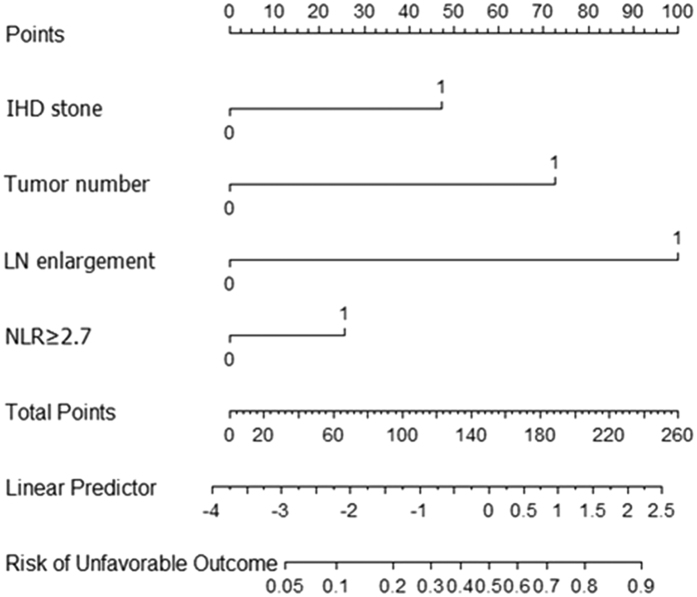
Nomogram for futile resection of intrahepatic cholangiocarcinoma in curative-intent surgery. LN, lymph node; NLR, neutrophil-to-lymphocyte ratio; IHD, intrahepatic duct.

After bootstrapping the samples and performing backward stepwise selection within each sample, the presence of IHD stones, tumor number, lymph node enlargement, and elevated NLR were selected for inclusion in the final model on the basis that they had the highest predictive accuracy (Fig. 1). The C-index for the original data was 0.847. The calibration curve showed good agreement between the prediction by the nomogram and actual observations, with the estimated slope of the calibration curve equal to 1.

### Nomogram of the validation cohort

The validation cohort consisted of 341 patients (Table 1). The median age was 62 years, and 222 (65.1%) patients were male. The median tumor size was 4.5 cm, and 47 (13.8%) patients had multiple tumors. Lymph node enlargement was positive in 107 (31.4%) patients. The median NLR was 2.0, and R2 resection occurred in 38 (11.1%) patients. The reasons for R2 resection were as follows: peritoneal seeding (*n* = 12), distant lymph node metastasis (*n* = 7; aortocaval (n = 2), cardiophrenic (n = 2), paraaortic (n = 3)), hilar or major vessel invasion (*n* = 6), multiple intrahepatic metastases in both lobes (*n* = 5), small remnant liver volume (*n* = 5), and abdominal wall invasion (*n* = 3). Among them, 15 patients (39.5%) underwent laparotomy only, while resection of the lesion was precluded. None of these findings were apparent in preoperative imaging studies.

**Figure 2:**
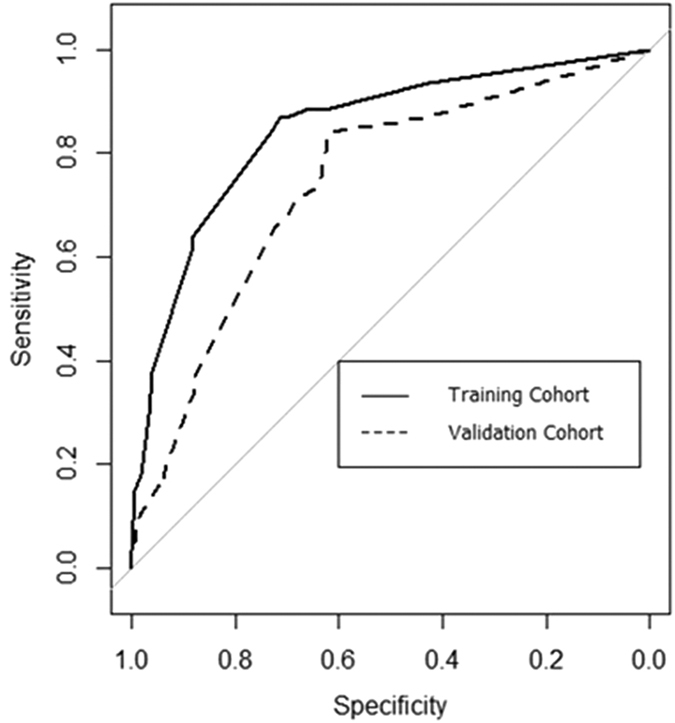
Discrimination and validation of nomogram. The area under the curve of the nomogram was 0.847 in the training cohort (linear line), and 0.740 in the validation cohort (dotted line).

The nomogram was applied to the validation cohort. The C-index of the validation cohort was 0.740, which was lower than that of the training cohort (Fig. 2).

### Predictive value of the proposed nomogram for unresectability in the validation cohort

When the prediction of futile resection in the proposed nomogram was stratified using the probability of futile resection based on the previous literature, the highest rate of futile resection was 36%^6^. At this point (128 points with a probability of futile resection of 36%), the positive and negative predictive values for futile surgery in the validation cohort was 65.4% and 93.3%, respectively.

## Discussion

Surgical resection offers the only chance of a cure in the case of IHCC. In this study, a novel preoperative nomogram to predict futile resection was developed based on a preoperative workup, and validation was performed. In the multivariate analysis of the training cohort data, the tumor number, lymph node enlargement, the presence of IHD stones, and an elevated NLR (≥2.7) showed a significant correlation with futile resection. These factors were all selected into the nomogram. The C-index of the nomogram for the training and validation cohorts was 0.847 and 0.740, respectively.

Complete resection of a tumor can be achieved in cases of resectable tumors and competent surgical techniques. In this study, highly experienced surgeons performed all the curative-intent surgeries. Thus, we could exclude surgical factors and focus on the preoperative prediction of futile surgery. Recent advances in imaging studies mean that a large amount of information about tumors can be obtained before surgical management^7^. However, discrepancies still occur between preoperative and intraoperative findings due to radiologically occult metastatic disease. Weber *et al*. report an R2 resection rate of 37.7% (20/53)^6^. This rate was much higher than that found in recent studies, which report an R2 resection rate of about 10%^8^,^9^, and it was also higher than that found in the present study (13.8%, 99/718). Such variations in R2 resection rates may be due to differences in resolution of the imaging modalities and advances in imaging studies. In the present study, the overall postoperative complication rate was similar between the R2 and non-R2 resection groups (11.1% vs.11.9%, *P = *0.777), whereas the R2 resection groups showed significantly longer median hospital stays than the non-R2 resection group (17 days vs. 14 days, *P* < 0.001), which can bring an additional burden to the patients and their families. To prevent unexpected R2 resection, the addition of a minimally invasive workup, such as staging laparoscopy^10^,^11^ or endoscopic ultrasound with fine-needle aspiration (EUS-FNA)^12^,^13^,^14^,^15^,^16^, can be used to identify unresectable IHCC by detecting metastatic lymph node or peritoneal metastasis, as well as locally advanced diseases (e.g., hilar or major vessel invasion and multiple intrahepatic metastases in both lobes).

Little is known about preoperative predictive markers of futile resection in IHCC patients undergoing curative-intent surgery. Studies to date have only investigated ways of predicting overall outcomes of patients with IHCCs^17^. Wang *et al*. suggest the use of a prognostic nomogram based on serum level of CEA and CA-19-9, vascular invasion, lymph node status, direct extrahepatic invasion, tumor number, and tumor size after surgical resection of IHCC^18^. In their study, the performance of the nomogram was better than that of a conventional staging system. Hyder *et al*. also develop a nomogram based on the patient’s age, tumor size, tumor number, lymph node status, vascular invasion, and underlying cirrhosis that predicted the overall survival of patients with resectable IHCC^19^. Jiang *et al*. describe a Fudan scoring system based on ALP, serum CA-19-9 level, tumor number, tumor size, and tumor boundary type, which proved useful for preoperative predictions of overall surgical outcomes^20^.

In the present study, it was assumed that the tumoral burden was strongly associated with unidentified metastasis in the conventional preoperative workup and that the tumoral burden would elevate the risk of futile resection by increasing the spread of local (e.g., hilar or major vessel invasion and multiple intrahepatic metastases in both lobes) or systemic (e.g., peritoneal seeding and distant lymph node metastasis) diseases. Some debates have focused on the preoperative diagnosis of lymph node metastasis. Adachi *et al*. point out poor sensitivity (50%) of preoperative evaluation of lymph node enlargement (>10 mm on CT or MR) for diagnosing lymph node metastasis, and in multivariate analysis, lymph node enlargement was not identified as a predictive risk factor of intrahepatic cholangiocarcinoma. Considering long-term outcomes, the authors suggest that preoperative lymph node enlargement should not determine to abandon radical resection. However, in our study, we focused on the short-term surgical outcomes and the proper decision making process using preoperative data prior to curative-intent surgical resection. In addition, in our proposed nomogram, lymph node enlargement only (100 points) is not always consistent with a high risk of unfavorable surgical outcome (≥128 points).

As the tumoral burden can only be detected on preoperative imaging studies before exploration, laboratory findings were included in the predictive model to reduce discrepancies between the preoperative and intraoperative findings. The NLR is the ratio between the absolute neutrophil count and absolute lymphocyte count in peripheral blood. An elevated NLR is associated with the presence of significant systemic inflammation, relative depletion of lymphocytes, and impairment of the host immune reaction against the tumor^21^,^22^. The preoperative NLR alone was reported to be a potent predictor of a poor prognosis in various gastrointestinal malignancies, such as colorectal cancer^23^, gastric cancer^24^, primary liver cancer^25^, pancreatic cancer^26^, and IHCC^27^. In the current study, a significant correlation was seen between an elevated NLR and futile resection in the training cohort, and it seemed to be a possible single predictor of IHCC.

A nomogram is a potent predictive model, which is intuitive and provides easily accessible information to physicians and surgeons. Nomograms were reported to be superior to conventional predictive models and staging systems^18^, such as the TNM staging system. One of the advantages of a nomogram is that it can quantify the overall patient risk and make it easy to compare the possibility of an unfavorable outcome according to different risk factors. For example, according to the predictive nomogram in the present study, a 65-year-old woman with a 7.0 cm-sized single IHCC in left lobe, positive lymph node enlargement, negative vascular invasion in imaging studies, the presence of IHD stones, and an NLR of 4.4, scored 173 points. Thus, the patient’s estimated probability of futile resection was 64% (Fig. 1). Based on the previous literature^6^, a level of 36% was assumed to represent the highest probability of futile resection. Thus, the aforementioned patient belonged to a high-risk group. This patient underwent curative intent surgery, but the tumor could not be completely removed (R2 resection) due to locally advanced tumor invasion. The probability of the nomogram, which included the tumor number, lymph node enlargement, the presence of IHD stones, and an elevated NLR, predicting futile resection in the training cohort was good (C-index of 0.847). However, its performance decreased in the validation cohort (C-index of 0.740). One possible reason for this is the differences between the baseline characteristics of the two cohorts (Table 1). Furthermore, as the cohorts were divided according to the year of the surgery, time intervals existed between the two cohorts. New advances in imaging studies, combined with the surgeon’s experience, might lead to the detection of more unresectable cases prior to surgical exploration in the validation cohort^8^. Although considerable differences were detected between the two groups in terms of baseline characteristics, the C-index of validation cohort was relatively consistent (≥0.7). This result suggests that our novel nomogram could be applicable to other cohorts in real clinical situations.

**Figure 3:**
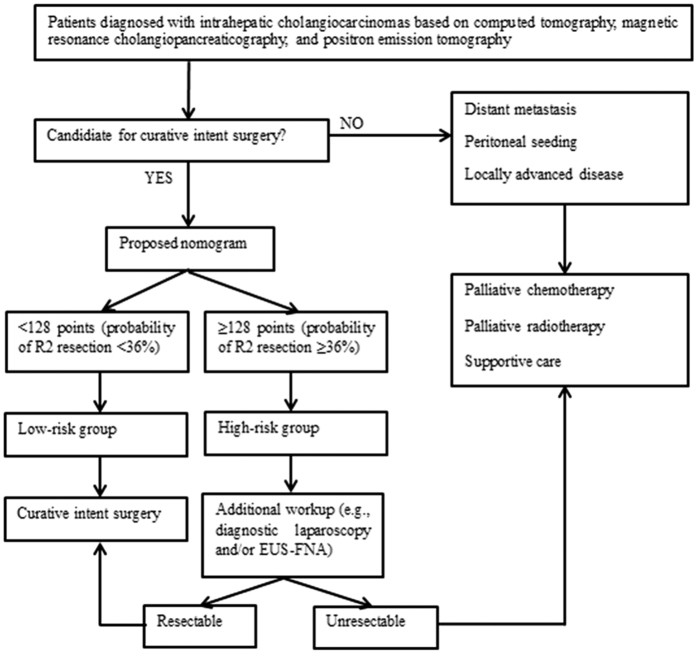
Proposed algorithm of the optimal cutoff for curative-intent surgery of intrahepatic cholangiocarcinoma. EUS-FNA, endoscopic ultrasound-guided fine needle aspiration.

Using this nomogram, we propose an algorithm for the treatment of IHCC (Fig. 3). If a patient’s estimated probability of futile resection in the proposed nomogram is higher than the highest level of futile resection in conventional surgical exploration (probability of futile resection of 36%), the patient is defined as belonging to the high-risk group, and an additional workup is recommended to confirm the unresectable state before surgical exploration. Based on a low positive predictive value (65.4%) and high negative predictive value (93.3%) of our nomogram, in the low-risk group, curative-intent surgery without any further evaluation may be acceptable, whereas further evaluations (staging laparoscopy or EUS-FNA) may be recommended to confirm the disease status before surgical exploration in the high-risk group with radiologically occult metastatic disease. The proposed algorithm can help avoid excessive preoperative workup in the low-risk group and unnecessary surgical exploration and longer hospital stays in the high-risk group. It can also reduce the workload of surgeons and improve the cost-effectiveness of the management of potentially resectable IHCC.

This study has some limitations. First, the nomogram was produced and validated using retrospective data from a single institute. Thus, there may be considerable selection bias. Furthermore, as the data were collected over 10 years, heterogeneity of the patient cohort may be present. Second, possible risk factors related to the recurrence of tumors and overall survival rates (e.g. growth type of the tumor) were not included in the present analysis. Third, although the proposed nomogram was validated, the validation cohort comprised patients from the same center. A multicenter-based external validation is needed to verify the performance of nomogram.

In conclusion, the accurate prediction of futile resection in IHCC before curative-intent surgery remains a challenge. The present study described the development of a simple and novel preoperatively applicable nomogram based on the tumor number, lymph node enlargement, the presence of IHD stones and elevated NLR (≥2.7) to predict unresectable IHCC. Our proposed nomogram indicated high-risk patients (≥128 points with a probability of futile resection of ≥36%) may undergo staging laparoscopy or EUS-FNA to determine the disease status before curative-intent surgery. Although the nomogram needs to be further validated, it may help physicians and surgeons to select appropriate treatment options and improve overall surgical outcomes.

## Methods

### Study design and patient cohort

The study cohort consisted of consecutive patients who were diagnosed with IHCC and underwent curative intent surgery between January 2005 and December 2014 at the Asan Medical Center. A routine clinical evaluation, laboratory tests, and a preoperative evaluation with routine imaging workup of IHCC, including a multichannel (32 or 64) computed tomography (CT) scan, magnetic resonance cholangiopancreatography (MRCP), or positron emission tomography-computed tomography (PET-CT), were performed to determine the presence of extrahepatic tumors. Routine studies included a chest roentgenogram, an upper endoscopy, and a colonoscopy. All imaging studies (CT scan, MRCP, or PET-CT in selected cases) were performed within 2 weeks before exploration.

Primary curative-intent surgery was carried out in cases regarded as resectable and operable. If the patient had multiple adjacent IHCCs in the same surgical field, it was deemed resectable. Patients who were clearly unresectable based on the imaging studies (e.g., distinct extrahepatic metastasis and peritoneal seeding) or inoperable (e.g., cardiovascular abnormality, respiratory abnormality, or poor general performance) were excluded and underwent palliative treatment or supportive care. The final diagnosis of IHCC was based on the pathological examination of the resected specimen or an intraoperative excisional biopsy. Patients who were diagnosed with hilar cholangiocarcinomas, hepatocellular carcinomas, or metastatic liver cancers were excluded. The study group was divided into a training cohort (early 6 years, 2005–2010) to build a predictive model, and a validation cohort (late 4 years, 2011–2014) to evaluate the performance of the predictive model. All patients provided written and informed consent before undergoing curative-intent surgery. All the examinations and surgeries were performed in accordance with relevant guidelines and regulations. This study was approved by the Institutional Review Board of Asan Medical Center in 2015.

### Definitions of preoperative variables and postoperative outcomes

Clinical information on the patients, tumoral characteristics, and surgical outcomes were extracted from the patients’ medical records. The patients’ age, gender, body mass index (BMI), underlying physical condition, and laboratory data, including tumor markers (carcinohydrate antigen [CA]-19-9 and carcinoembryonic antigen [CEA]), platelet count, and NLR, were analyzed^21^,^27^. An elevated preoperative NLR was considered representative of host inflammation. NLR was calculated within 7 days before exploration. The tumoral characteristics analyzed included the tumor size, tumor number, tumor location, vascular invasion, and lymph node enlargement based on imaging studies at the time of diagnosis. The size of the tumor was calculated by measuring the longest length of the tumor (the largest tumor in the case of multiple tumors). The number of tumors was classified as single or multiple (≥2). Lymph node enlargement was defined as lymph nodes with a diameter of ≥10 mm in imaging studies^12^.

BMI cut-off value for obesity was 25 kg/m^2^. Diabetes mellitus was defined as a fasting blood glucose ≥126 mg/dl or treatment with diabetes medication. Hypertension was defined as blood pressure ≥140/90 mmHg or treatment with anti-hypertensive medications. Metabolic syndrome was defined as a cluster of three or more of the following conditions: abdominal obesity (waist circumference >102 cm [male] or >88 cm [female]); serum triglyceride ≥150 mg/dl; high density lipoprotein cholesterol <40 mg/dl (male) or <50 mg/dl (female); blood pressure ≥130/85 mmHg; and fasting serum glucose ≥110 mg/dl. Alcohol consumption indicated the patient drank over 210 g (male) or 140 g (female) of alcohol per week. Liver cirrhosis and fatty liver disease were defined based on characteristic findings in imaging studies. Clonorchiasis infestation was defined based on characteristic findings in the intrahepatic duct or positive serological test results.

Curative-intent surgery is defined as the removal of all suspected tumors and metastatic lymph nodes in the surgical field. All the surgeries were performed by highly experienced surgeons at the Asan Medical Center. The surgical outcomes were categorized as R0 (complete removal of the tumor with a negative histological resection margin), R1 (grossly complete resection but microscopic tumor involvement in the histological resection margin), and R2 (incomplete removal of the tumor and grossly remnant disease) resection. In this study, R0 and R1 resections were regarded as favorable outcomes (macroscopic curative resection). R2 resection was considered an unfavorable outcome and was defined as a composite outcome of a macroscopic incomplete resection, such as peritoneal seeding, intrahepatic metastasis, or locally advanced tumor invasion. Unexpected R2 resection upon surgical exploration was defined as futile resection because it could not benefit from a surgical approach and was delayed following palliative therapy. If the patient underwent several operations for recurrent IHCCs during the study period, the data from the first surgery were used.

### Nomogram of the training cohort

To predict the probability of futile resection in the training cohort, univariate, and multivariate logistic regression analyses were performed. Significant factors from the logistic regression analysis and other potent risk factors were used to develop the predictive nomogram. The predictive accuracy of the final model was based on its discrimination ability and calibration curves.

To validate the predictive model (i.e., detect evidence of overfitting), bootstrapping was performed. By bootstrapping and backward stepwise selection of the variables in the resampled data, the variables with the highest predictive accuracy were selected for inclusion in the final model. To estimate the predictive accuracy of the model, the bias-corrected concordance index (C-index) was used. All internal validations of the model were performed using 1,000 bootstrap samples.

### Nomogram of the validation cohort

Clinical information on the patients, tumoral characteristics, and surgical outcomes were extracted and used as a validation dataset. The C-index for futile resection was calculated using this dataset.

### Statistical analysis

All the statistical analyses were performed using SPSS version 20 (SPSS statistics, Armonk, NY) and R version 3.0.2 (http://www.r-project.org). Categorical variables were described as frequencies and percentages (%), and were compared using the chi-squared test or Fisher’s exact test for the component. Continuous variables were described as the mean and standard deviation or the median and interquartile range, and they were compared using a Student’s t-test or Mann-Whitney test for the component. A *P* value of less than 0.05 was defined as statistically significant.

## Additional Information

**How to cite this article**: Nam, K. *et al*. Novel Preoperative Nomogram for Prediction of Futile Resection in Patients Undergoing Exploration for Potentially Resectable Intrahepatic Cholangiocarcinoma. *Sci. Rep.* **7**, 42954; doi: 10.1038/srep42954 (2017).

**Publisher's note:** Springer Nature remains neutral with regard to jurisdictional claims in published maps and institutional affiliations.

## Figures and Tables

**Table 1:** Clinical characteristics of the training and validation cohort.

Variables	Training cohort		Validation cohort		*P* value
	(n = 377)		(n = 341)		
Age, median (IQR)	60 (53–66)		62 (56–69)		0.005
Gender, male	260	69.0%	222	65.1%	0.271
BMI, kg/m^2^, median (IQR)	23.3 (21.6–25.4)		23.7 (21.9–25.6)		0.159
Diabetes	97	25.7%	65	19.1%	0.033
Hypertension	113	30.0%	129	37.8%	0.026
Metabolic syndrome	19	5.0%	5	1.5%	0.008
Underlying liver condition					
Hepatitis B	70	18.6%	73	21.4%	0.341
Hepatitis C	8	2.2%	4	1.2%	0.301
Alcohol consumption	83	22.0%	100	29.3%	0.025
Liver cirrhosis	30	8.0%	50	14.7%	0.004
Fatty liver disease	10	2.7%	5	1.5%	0.267
IHD stones	30	8.0%	21	6.2%	0.349
Clonorchiasis infestation	61	16.2%	34	10.0%	0.014
PSC	1	0.3%	0	0%	1.000
Tumor size, cm, median (IQR)	5.0 (3.3–7.5)		4.5 (3.2–6.5)		0.138
Tumor location					0.500
Right lobe	168	44.6%	164	48.1%	
Left lobe	179	47.5%	147	43.1%	
Both lobe (central)	30	8.0%	30	8.8%	
Tumor number, ≥2	45	11.9%	47	13.8%	0.460
Vascular invasion	89	23.6%	90	26.4%	0.389
Lymph node enlargement	134	35.5%	107	31.4%	0.238
Laboratory data, median (IQR)					
Neutrophil, %	60.8 (53.1–69)		58.9 (51.5–66.3)		0.009
Lymphocyte, %	27.6 (19.7–34.3)		29.0 (22.4–36.5)		0.004
Platelet, 10^3^/uL	232 (185–290)		216 (171–271)		0.005
Albumin, g/dL	3.7 (3.4–4)		3.7 (3.4–4)		0.295
CRP, mg/dL	0.2 (0–0.9)		0.2 (0–0.9)		0.898
AST, U/L	26 (22–37)		27 (21–34.5)		0.851
ALT, U/L	22 (16–35)		23 (16–37)		0.510
ALP, IU/L	110 (80.5–165)		96 (70–154)		0.005
GGT, IU/L	69 (34–155)		67 (31–158)		0.847
Total bilirubin, mg/dL	0.8 (0.6–1)		0.6 (0.5–0.9)		<0.001
Direct bilirubin, mg/dL	0.2 (0.2–0.3)		0.2 (0.2–0.3)		0.898
CEA, ug/L	1.9 (1–5)		2.8 (1.7–4.9)		<0.001
CA-19-9, U/L	32.5 (8.2–202.5)		38.1 (10.5–340.3)		0.106
NLR, median (IQR)	2.2 (1.6–3.6)		2.0 (1.4–3)		0.005
Surgical outcome					0.075
R0	239	63.4%	241	70.7%	
R1	77	20.4%	62	18.2%	
R2	61	16.2%	38	11.1%	
Postoperative complication	43	11.4%	31	9.1%	0.308
Biloma, bile leakage, stricture	11		5		
Wound dehiscence	10		2		
Complicated fluid collection	6		10		
Infection, abscess	6		8		
Postoperative bleeding	5		4		
Postoperative adhesion	2				
Bowel perforation	1				
Portal vein stenosis	1				
Pulmonary thromboembolism	1				
Respiratory failure			1		
Acute liver failure			1		
Status					<0.001
Alive	95	25.2%	166	48.7%	
Dead	282	74.8%	175	51.3%	

ALP, alkaline phosphatase; ALT, alanine aminotransaminase; AST, aspartate aminotransferase; BMI, body mass index; CA-19-9, carcinohydrate antigen 19-9; CEA, carcinoembryonic antigen; CRP, C-reactive protein; GGT, gamma-glutamyl transpeptidase; IQR, interquartile range; NLR, neutrophil-to-lymphocyte ratio; PSC, primary sclerosing cholangitis; SD, standard deviation.

**Table 2:** Univariate and multivariate analysis of the training cohort.

Variables	Univariate analysis			Multivariate analysis		
	OR	95% CI	*P* value	OR	95% CI	*P* value
Age, ≥65	0.841	0.453–1.508	0.571			
Gender, male	1.430	0.799–2.515	0.220			
BMI, ≥25 kg/m^2^	0.770	0.398–1.418	0.417			
Metabolic syndrome	1.926	0.603–5.260	0.226			
Alcohol consumption	1.321	0.687–2.443	0.387			
Liver cirrhosis	0.783	0.225–2.105	0.660			
Fatty liver disease	1.305	0.194–5.368	0.740			
**IHD stones**	**2.902**	**1.240–6.435**	**0.010**	**3.464**	**1.233–9.528**	**0.016**
Clonorchiasis infestation	0.506	0.136–1.802	0.290			
Tumor size, ≥5.0 cm	1.075	0.991–1.163	0.074			
**Tumor location**, **central**	**1.817**	**1.182–2.805**	**0.007**			
**Tumor number**, **≥2**	**5.678**	**2.883–11.142**	**<0.001**	**5.987**	**2.643–13.887**	**<0.001**
**Vascular invasion**	**2.096**	**1.151–3.751**	**0.014**			
**LN enlargement**	**12.554**	**6.463–26.460**	**<0.001**	**12.655**	**6.243–27.947**	**<0.001**
AST	1.002	0.993–1.010	0.704			
ALT	1.006	0.997–1.013	0.171			
**ALP**	**1.002**	**1.000–1.004**	**0.042**			
Total bilirubin	1.141	0.839–1.503	0.336			
Albumin, <3.5	1.691	0.953–2.965	0.069			
CRP, ≥1.0	1.419	0.756–2.577	0.261			
**NLR**, **≥2.7**	**2.106**	**1.209–3.677**	**0.008**	**1.981**	**1.027–3.850**	**0.042**
**CA-19–9**, **≥37**	**1.889**	**1.086–3.340**	**0.026**			

ALP, alkaline phosphatase; ALT, alanine aminotransaminase; AST, aspartate aminotransferase; BMI, body mass index; CA-19-9, carcinohydrate antigen, CI, confidence interval; CRP, C-reactive protein; IHD, intrahepatic duct; LN, lymph node; NLR, neutrophil-to-lymphocyte ratio; OR, odds ratio.
